# ERRα protein is stabilized by LSD1 in a demethylation-independent manner

**DOI:** 10.1371/journal.pone.0188871

**Published:** 2017-11-30

**Authors:** Julie Carnesecchi, Catherine Cerutti, Jean-Marc Vanacker, Christelle Forcet

**Affiliations:** Institut de Génomique Fonctionnelle de Lyon, Université de Lyon, Université Lyon I, CNRS UMR5242, Ecole Normale Supérieure de Lyon, Lyon, France; Roswell Park Cancer Institute, UNITED STATES

## Abstract

The LSD1 histone demethylase is highly expressed in breast tumors where it constitutes a factor of poor prognosis and promotes traits of cancer aggressiveness such as cell invasiveness. Recent work has shown that the Estrogen-Related Receptor α (ERRα) induces LSD1 to demethylate the Lys 9 of histone H3. This results in the transcriptional activation of a number of common target genes, several of which being involved in cellular invasion. High expression of ERRα protein is also a factor of poor prognosis in breast tumors. Here we show that, independently of its demethylase activities, LSD1 protects ERRα from ubiquitination, resulting in overexpression of the latter protein. Our data also suggests that the elevation of LSD1 mRNA and protein in breast cancer (as compared to normal tissue) may be a key event to increase ERRα protein, independently of its corresponding mRNA.

## Introduction

Lysine Specific Demethylase 1 (LSD1 *aka* KDM1A) is an enzyme that removes mono- or dimethyl groups from Lys 4 or Lys 9 of histone H3 (H3K4, H3K9, respectively), leading to transcriptional repression or activation, respectively [1–2]. The choice between these two types of activities is apparently dictated by the transcriptional (co)-factors with which LSD1 interacts. For instance, LSD1 mostly behaves as a transcriptional repressor when interacting with CoREST [3]. In contrast, the Androgen Receptor or an Estrogen Receptor-PELP1 complex can, at least in part, switch LSD1 activities towards transcriptional activation [2, 4–6]. In addition to H3, LSD1 also displays non-histone substrates and its activities lead to various outcomes. Indeed, LSD1 demethylates K370 residue on p53, which prevents the latter to interact with its co-activator 53BP1, thereby eventually leading to inhibition of p53 activity [7]. In addition, demethylation of non-histone substrates by LSD1 can impact on protein stability. For instance, LSD1-driven demethylation of Dnmt1 or of E2F1 increases their stability [8–9], whereas demethylation of Mypt1 induces its degradation [10]. LSD1 is strongly expressed in various types of cancers, including from the prostate and the breast [11–14], suggesting an active role in promoting traits of cancer progression. In this line, a number of reports have indeed indicated that LSD1 regulates various oncogenic processes, such as enhanced cell motility or metabolic reprograming (reviewed in [15]).

Estrogen-Related Receptor α (ERRα) is a member of the nuclear receptor (NR) family and, as such, acts a transcriptional regulator. In contrast to several other members of the NR family, no natural ligand has been, to date, identified for ERRα, which is thus referred to as “orphan” [16]. Work from various laboratories has indicated that this receptor promotes, amongst others, such processes as cellular migration and invasion, resistance to hypoxia, as well as metabolic reprograming, which all contribute to cancer aggressiveness [17–21] (reviewed in [22]). Interestingly, the expression of ERRα is strongly enhanced in several types of cancers as compared to the corresponding normal tissue [23–28] (reviewed in [29]). Several mechanisms have been proposed to account for this increased expression, ranging from local genomic amplification, effect of a transcriptional auto-regulatory loop as well as intervention of specific microRNAs [30–33]. However the possibility that stabilization of the ERRα protein may act as a possible process has not been addressed.

Recent work from our laboratory has shown that ERRα interacts with LSD1 and induces H3K9 demethylase activity on the latter [34]. ERRα and LSD1 display a number of common target genes that they regulate through H3K9 demethylation at the level of the transcriptional start site. Strikingly, these target genes are strongly enriched for gene-ontology terms related to cell migration and invasion, suggesting that both factors are together involved in cancer progression. Here we show that LSD1 overexpression protects ERRα from proteasome-dependent degradation. This activity, resulting in an increased receptor half-life, does not depend on LSD1-mediated demethylation of ERRα. Conversely genetic or pharmacologic inactivation of LSD1 results in decreased ERRα stability. Our data mining analysis suggests that elevation of LSD1 protein expression in breast cancer may be a key factor leading to increased ERRα protein level.

## Materials and methods

### Cell culture and transfections

HeLa and MDA-MB231 cells were cultured in DMEM supplemented with 10% FCS, 10U/ml penicillin and 10μg/ml streptomycin. For siRNA transient transfection, 3 10^5^ cells per ml were seeded in 6-well plate and 25pmol/ml of siRNAs against LSD1 (Invitrogen), ERRα (Dharmacon and Invitrogen) or control (medium GC Stealth RNA interference negative control duplexes, Invitrogen) (Table 1) were transfected with INTERFERin (Polyplus Transfection) according to the manufacturer’s protocol. Plasmid transfections were performed with Exgen500 (Euromedex) for HeLa cells and JetPRIME (Polyplus Transfection) for MDA-MB231 cells. LSD1-K661A mutant was generated by recombinant PCR and verified by sequencing. pSG5-flagERRαΔA/B and pSG5-flagERRαΔA/BΔAF2 have been described elsewhere [35]. Cells were harvested 48 hours after transfection. Cycloheximide (Sigma-Aldrich) was used at 50–100μg/ml, MG132 (Sigma-Aldrich) at 50μM, pargyline (Sigma) at 3mM and tranylcypromine (Sigma) at 200μM.

**Table 1:** Oligonucleotides used in this study.

For mRNA expression		
36b4	GTCACTGTGCCAGCCCAGAA	TCAATGGTGCCCCTGGAGAT
LSD1 (KDM1A)	ACCACAACAGACCCAGAAGG	CTCGGTGGACAAGCACAGTA
ERRα (ESRRA)	CAAGCGCCTCTGCCTGGTCT	ACTCGATGCTCCCCTGGATG
siRNAs		
ERRα#1	GGCAGAAACCUAUCUCAGGUU	CCUGAGAUAGGUUUCUGCCUC
ERRα#2	GAAUGCACUGGUGUCACAUCUGCUG	CAGCAGAUGAGACACCAGUGCUUC
LSD1#1	ACUUUGUAACUGUCGAGCUGC	GCAGCUCGACAGUUACAAAGU
LSD1#2	CCACGGAGCGACAGAGCGAGC	GCUCGCUCUGUCGCUCCGUGG

### Protein analysis

For co-immunoprecipitation assays, cells were harvested in Phosphate Buffered Saline (PBS) and pellets were resuspended in NP40 buffer (20mM Tris pH7.5, 150mM NaCl, 2mM EDTA, 1% NP40) supplemented with protease inhibitor cocktail (Sigma-Aldrich). 800μg to 1mg of proteins were pre-cleared for 2h on Sepharose-protein A (GE-Healthcare) and 3μg of antibodies were added for 4h at 4°C with rotation (ERRα, PP-H5844-00, R&D). Beads were then added to the extract and incubated for 1h, washed 5 times with NP40 buffer and finally resuspended in Laemmli buffer for immunoblotting analysis. 50μg of whole cell lysate were analysed as input fraction.

For western blot analysis, cells were lysed in NP40 or RIPA buffer supplemented with Protease Inhibitor Cocktail (Sigma-Aldrich). Proteins (25–50μg) were resolved on 8 to 15% SDS-PAGE, blotted onto PVDF membrane (GE-Healthcare) and probed with specific antibodies after saturation. The antibodies (and their dilution) used in this study were: ERRα (GTX108166, Genetex, 1/5000), hsp90 (API-SPA-830, Enzo Life Sciences, 1/3000), LSD1 (ab17721, Abcam, 1/1000), flag-M2 (F3165, Sigma, 1/3000), β-actin (A5060, Sigma, 1/10,000), myc (MMS-150R, Covance, 1/3000), AR (sc-13062, Santa Cruz, 1/1000 for western blot; sc-7305, Santa Cruz for immunoprecipitation).

### Analysis of ubiquitination

Protocol was adapted from [36]. Briefly, cells plated in 100mm dishes were harvested in 5ml of PBS whose 1/5 of the extract was used for whole cell lysate quantification analysis and the rest was centrifuged and resuspended in lysis buffer (6M Guanidine Hydrochloride, 10mM Tris pH8, 100mM phosphate buffer). Lysates were sonicated and supplemented with 5mM β-mercaptoethanol and 5mM imidazole. After centrifugation 100μl of Nickel affinity resins were added (His60 Ni Superflow, Clontech) and the lysate fractions were incubated overnight at 4°C under mild rotation. Nickel beads were washed 1x with lysis buffer, 1x with wash buffer pH8 (8M Urea, 10mM Tris pH8, 100mM phosphate buffer pH8), 3x with wash buffer pH6.3 (8M Urea, 10mM Tris pH6.3, 100mM phosphate buffer pH6.3). For washing steps, buffers were supplemented with 0.1% Triton and 5mM β-mercaptoethanol. Beads were resuspended with elution buffer (200mM Imidazole, 150mM Tris pH6.7, 5% SDS, 30% Glycerol, 720mM β-mercaptoethanol, 0,0025% bromophenol blue) and incubated at 30°C for 20min at 350rpm and boiled 2min at 95°C. Phosphate buffer pH8 and pH6.3 were prepared from a mixture of Na_2_HPO_4_ and NaH_2_PO_4_ buffers at 0.2M at appropriate ratio.

### RNA extraction and real-time PCR

Total RNAs were extracted using TriPure kit (Roche). 1μg of RNA was converted to first strand cDNA using IScript cDNA synthesis kit (Biorad). Real-time PCR were performed in a 96-well plate using the IQ SYBR Green Supermix (Biorad). Data were quantified by the ΔΔ-Ct method and normalized to 36b4 expression (Table 1).

### Bio-informatical analysis

RNA-seq (Illumina HiSeq) data obtained in human breast samples were collected from The Cancer Genome Atlas (TCGA) data portal in 2016/05 (https://gdc-portal.nci.nih.gov/). Data were available as raw counts or TPM (transcript per million) for a total of 20531 genes. Paired samples from 112 patients, made on one breast tumor and one normal sample from the same patient, were used to study gene expression.

### Statistical significance and quantifications

Statistical analyses were performed with Student t-test. Protein expression levels were quantified with ImageJ software and analysis revealed the ratio of the protein of interest related to a housekeeping gene (actin or hsp90).

## Results

### LSD1/ERRα mRNA expression in breast tumors

**Fig 1:**
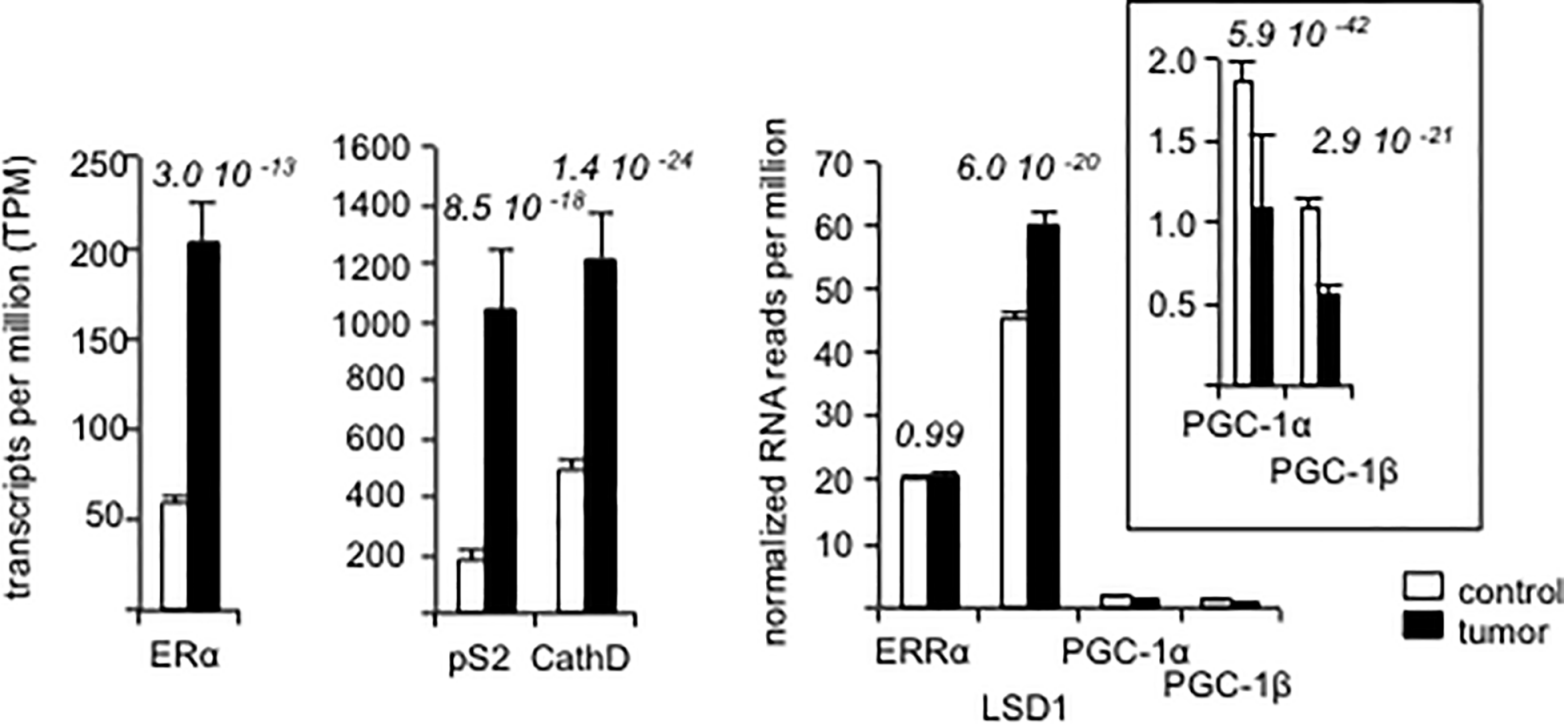
Gene expression in human breast samples. Differential expression between tumor and normal paired samples (n = 112) of the TCGA database was tested with the R DESeq2 package using raw counts. For each gene, the statistical significance was assessed by a p-value adjusted for multiple comparisons. Expression is given as TPM (transcript per million) and data are mean +/- sem.

Both LSD1 and ERRα proteins have been shown to display increased expression in cancer lesions as compared to the corresponding normal tissues including in breast tumors [11–14, 23–29]. However, it is unclear whether these elevations are due to increased expressions of the corresponding mRNAs. To examine this hypothesis, we analyzed data from a publicly available database (The Cancer Genome Atlas [TCGA]) for LSD1 (KDM1A) and ERRα (ESRRA) mRNA expression. We focused on data obtained by RNA-sequencing of pair-wise comparisons of breast cancer *vs* normal tissues within individuals, ending up in examination of 112-paired sequencing results. As expected, the expressions of ERα (ESRA) as well as those of its target genes pS2 (TFF1) and cathepsin D (CTSD) were more elevated in tumors than in normal tissues (Fig 1). In contrast, the expression of ERRα was not significantly altered in cancer samples suggesting that the elevation of ERRα protein in tumors does not involved an increase in the corresponding mRNA expression. However, we observed that LSD1 corresponding mRNA was increased in tumors as compared to normal tissues. These observations support the hypothesis that an elevation of LSD1 mRNA in tumors leads to increased LSD1 protein expression as demonstrated by others [11–14]. LSD1 and ERRα proteins interacting together, it is possible that an increase in LSD1 protein enhances ERRα protein levels through stabilization, without affecting the corresponding mRNA expression. In contrast, the expression of two other ERRα-interacting partners (PGC-1α [PPARGC1A] and PGC-1β [PPARGC1B]) was very weak and decreased in tumors *vs* control tissues. An involvement of the PGC-1 proteins in the increase of ERRα protein in tumors is thus unlikely.

### Inhibition of LSD1 results in decreased ERRα protein levels

**Fig 2:**
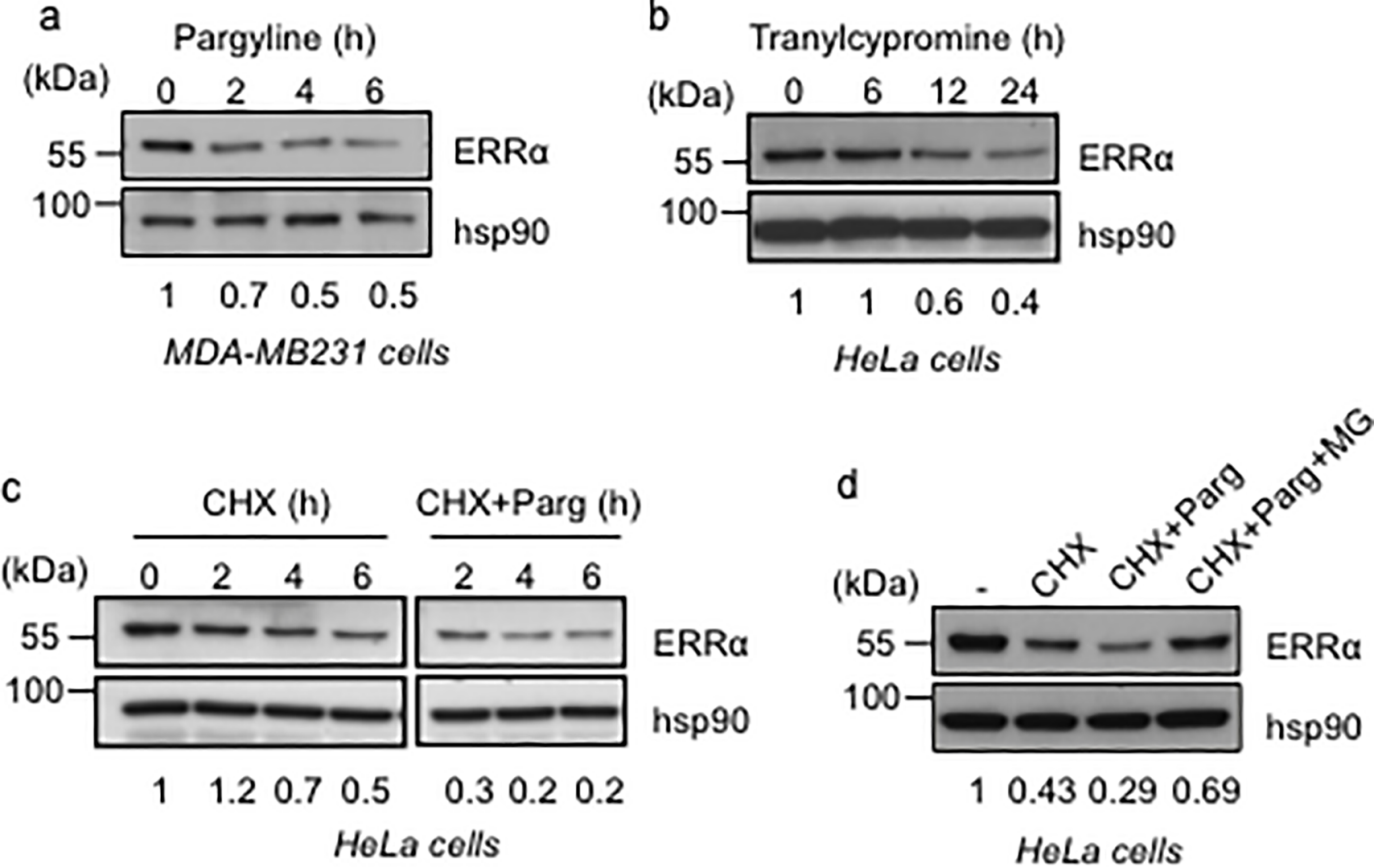
Exposure to mono-oxidase inhibitors reduces ERRα half-life. **a.** Expression of ERRα protein in MDA-MB231 cells after treatment with pargyline for the indicated time. **b.** Expression of ERRα protein in HeLa cells upon treatment with tranylcypromine for the indicated time. **c.** Steady state level of ERRα protein in HeLa cells was analyzed upon cycloheximide (CHX) treatment (50 μg/ml) for the indicated time, in the presence of Pargyline (Parg) or vehicle. **d.** Analysis of ERRα protein levels in HeLa cells after treatment with CHX, pargyline and MG132. Western blots were performed with the indicated antibodies. Quantification of ERRα levels (relative to hsp90) is displayed. Experiments were performed three times.

To examine the above hypothesis, we treated MDA-MB231 cells with pargyline, an inhibitor of monoamine oxidase (MAO) enzymes, including LSD1 [2, 37]. This resulted in a decrease in the apparent ERRα protein level (Fig 2A). Similarly, treatment of HeLa cells with tranylcypromine, another inhibitor of MAO enzymes, including LSD1 [37–38], also reduced the amount of ERRα protein, although with a different kinetic (Fig 2B). We next examined the half-life of ERRα protein by treating HeLa cells with cycloheximide (CHX), an inhibitor of protein synthesis. This half-life was dramatically decreased upon co-treatment with pargyline (Fig 2C). The effect of the compound was blunted, at least partially, by treatment with MG132, a proteasome inhibitor (Fig 2D), altogether showing that pargyline treatment impacted on ERRα protein stability, rather than synthesis.

**Fig 3:**
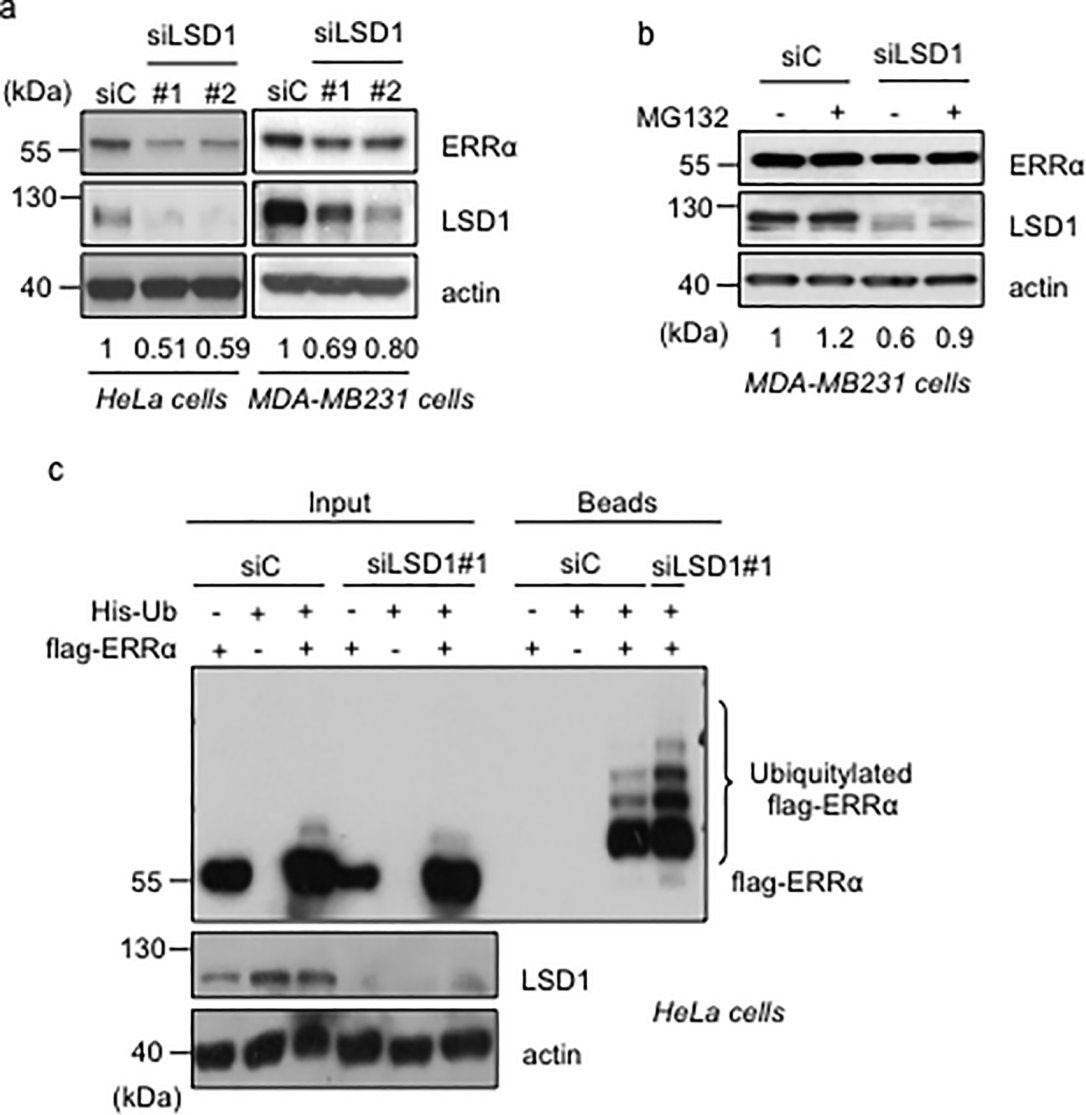
LSD1 stabilizes ERRα in a proteasome-dependent manner. **a.** Analysis of ERRα protein levels in the indicated cells after siRNA treatment. **b.** Analysis of ERRα and LSD1 protein levels in MDA-MB231 cells after siLSD1 treatment with supplementation with MG132 or vehicle. **c.** Detection of ERRα ubiquitylation in HeLa cells transfected with histidine-ubiquitine (His-Ub), flag-ERRα and the indicated siRNA. Beads represent the purified fraction. Ubiquitylated ERRα was detected using flag antibody. Quantification of ERRα levels (relative to actin) is displayed (**a, b**).

The effects of pargyline and tranylcypromine suggest that LSD1 is involved in ERRα stabilization. However both compounds inhibit LSD1 in a non-specific manner and display additional targets. To examine the impact of LSD1 on ERRα stability, we knocked down LSD1 using two different siRNAs. In both HeLa and MDA-MB231 cells, these treatments decreased the level of ERRα protein (Fig 3A). A time-dependent effect of siLSD1s was also observed on ERRα in HeLa cells (S1A Fig). In both cell types, these effects were rescued by inhibition of the proteasome through MG132 treatment (Fig 3B and S1B Fig), indicating that LSD1 acts on ERRα in a post-transcriptional manner. Consistently, siLSD1 treatment did not result in any variation of the level of ERRα mRNA (S1C Fig). The capacity of MG132 to rescue the effect of siLSD1 treatment on ERRα stability indicates an involvement of the proteasome. This also suggests that LSD1 protects ERRα from ubiquitination. This hypothesis was tested using co-transfection of a flag-tagged ERRα construct together with a His-tagged ubiquitin plasmid. Our data indicate that siRNA-mediated LSD1 depletion results in increased ubiquitination of transfected ERRα (Fig 3C and S1D Fig).

### ERRα protein is stabilized by LSD1 in a demethylation-independent manner

**Fig 4:**
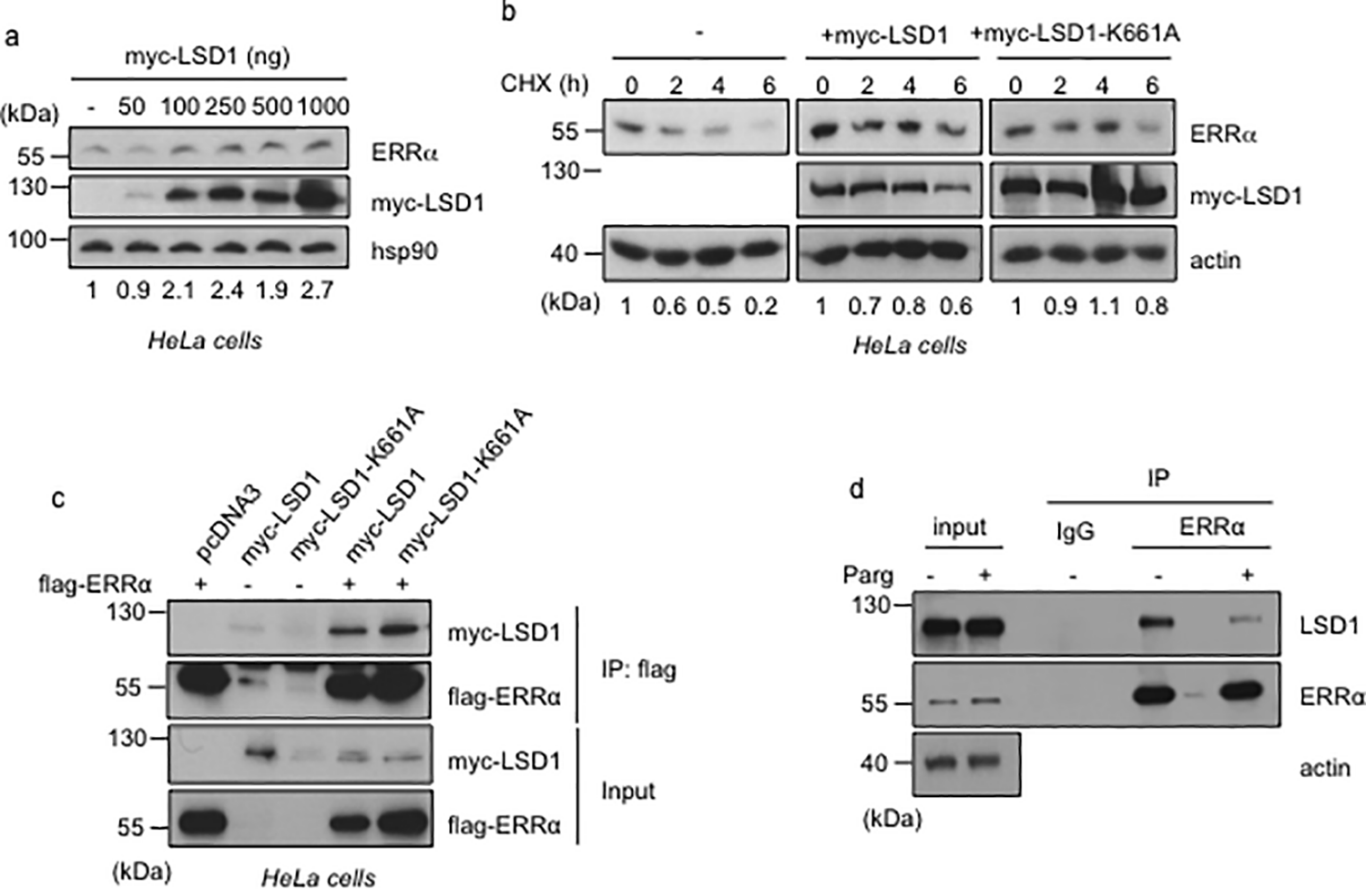
ERRα is stabilized by LSD1 independently of the latter’s enzymatic activity. **a.** Expression of ERRα protein in HeLa cells transfection with the indicated amount of myc-tagged LSD1. **b.** Steady state level of endogenous ERRα protein in HeLa cells transfected with myc-tagged wild type or catalytic mutant (K661A) LSD1, after CHX treatment (100 mg/ml) for the indicated time. Western blots were probed with the indicated antibodies and quantification of ERRα levels (relative to hsp90 or actin) is displayed. **c.** HeLa cells were transfected with flag-ERRα and together with myc-tagged LSD1 derivatives as indicated above. Co-immunoprecipitations were performed using flag antibody. flag-ERRα and myc-LSD1 were detected using the corresponding antibodies. **d.** Co-immunoprecipitation of endogenous proteins with anti-ERRα antibody (or rabbit IgG as control) from HeLa cells treated with pargyline or vehicle (-) in the presence of MG132.

Our results above indicate that pharmacological or genetic inhibition of LSD1 results in a decrease of ERRα stability. We next examined whether overexpression of LSD1 is capable of stabilizing ERRα. To this end, increasing amounts of a tagged LSD1 (myc-LSD1) plasmid were transfected into HeLa cells. This resulted in a dose-dependent elevation of the endogenous ERRα protein levels (Fig 4A). To determine whether this effect was active at the post-transcriptional level, we blocked protein synthesis using CHX. Transfection of myc-LSD1 also resulted in increased ERRα protein levels under these conditions (Fig 4B), indicating an effect on ERRα protein stability. We next examined whether the stabilizing effect of LSD1 depends on its demethylase activity. To this end an LSD1 construct mutated in its enzymatic pocket (LSD1-K661A, [3]) was transfected in HeLa cells. As in the case of wild type LSD1, overexpression of the catalytically inactive LSD1 led to increased endogenous ERRα protein levels (Fig 4B). As a control we verified by co-immunoprecipitation that both wild type and mutant LSD1 proteins interacted with co-transfected ERRα (Fig 4C). Together this indicates that the enzymatic activity of LSD1 is not involved in its capacity to stabilize ERRα protein. This statement is in apparent contradiction with our data above (Fig 2), demonstrating that treatment with inhibitors of LSD1 activity results in ERRα destabilization. In fact, co-immunoprecipitation experiments (performed in the presence of MG132 to prevent ERRα degradation) indicated a strong reduction in ERRα-LSD1 interactions upon pargyline or tranylcypromine treatments (Fig 4D and S1E Fig). This suggests that these compounds here merely act as a disruptor of ERRα-LSD1 physical contacts, rather than as inhibitors of LSD1 activity *per se*. We thus concluded that the demethylase activity of LSD1 is not involved in its ERRα-stabilizing effect.

## Discussion

In a previous report, we had shown that the LSD1 histone demethylase physically interacts with ERRα [34]. Here we demonstrate that LSD1 protects the ERRα protein from proteasome-dependent degradation, leading to increased receptor half-life. Conversely, LSD1 inactivation leads to reduced receptor levels. This effect has a certain level of specificity since inactivation of LSD1 does not destabilize the Androgen Receptor (S2A Fig), although both proteins interact in MDA-MB231 cells (S2B Fig). On another hand, siRNA-mediated ERRα inactivation does not impact on LSD1 stability (S2C Fig), indicating that the protective effect is not reciprocal. Interaction of ERRα with transcriptional co-activators such as PGC-1α depends on the AF2 transactivation domain, which forms the extreme C-terminal part of ERRα [39]. In contrast, interaction with LSD1 does not require this domain or the putative AF1 transactivation domain, which is located on the N-terminal part (A/B domain) of the receptor [34]. Consistently, the use of ERRα mutants shows that stabilization by LSD1 is still exerted when both transactivation domains are deleted (S2D Fig). A decrease in the half-life of ERRα was observed upon both pharmacological and genetic inactivation of LSD1. However, the effect of LSD1 does not depend on its capacity to demethylate ERRα. Indeed, a catalytically-dead LSD1 mutant, which retains the capacity to interact with ERRα, is still able to impact on the receptor’s stability. In addition, although pargyline (which otherwise blocks LSD1 enzymatic activity) induces ERRα destabilization, its primary effect here appears to disrupt the interaction between LSD1 and ERRα. Altogether this suggests that LSD1 here merely acts as steric hindrance, preventing yet-unidentified ubiquitin ligase(s) from accessing to critical domains of ERRα. This is in contrast with other situations in which LSD1 modulates the half-life of various proteins either positively (*e*.*g*. Dnmt1, E2F1) or negatively (*e*.*g*. Mypt1) through their demethylation [8–10]. However, our data do not exclude the possibility that ERRα may undergo methylation, an event that could be necessary to recognition and thus protection by LSD1.

Several reports have documented an increased ERRα expression in diverse types of cancers [23–28], reviewed in [29]). Various mechanisms have been proposed to account for such a phenomenon. For instance, a genomic amplification of the ERRα locus in squamous cell carcinoma has been demonstrated [31]. A regulatory loop has also been documented in which the ERRα protein binds to discrete response elements in the vicinity of its corresponding promoter and auto-induces its expression through a feed-forward mechanism [30]. However, this mechanism does not appear active in all cells. Indeed, in MCF7 cells, induction of ERRα protein degradation by its inverse agonist XCT790 does not impact on ERRα-corresponding mRNA, which is expected in an auto-regulatory loop scheme [40]. Regulations of ERRα expression at the post-transcriptional level have also been documented. For instance, several microRNAs (miRs) induce the degradation of ERRα-corresponding mRNA leading to reduced protein levels [32–33, 41]. Interestingly, at least some of these miRs (miR-135a, miR-497) display a reduced expression in cancer (*i*.*e*. opposite to ERRα) [33, 42], together suggesting that this reduction may account for the increase in ERRα expression at the mRNA level. Nevertheless, our analysis of publicly available database suggests that ERRα mRNA is equally expressed in breast cancer *vs* normal tissues. It should be noted that the study presented here is very limited, focusing on available 112 pair-wise comparisons, therefore preventing the establishment of a definitive conclusion. Setting aside these reservations allows us to propose that protein stabilization may be an important mechanism to increase ERRα expression in cancer *vs* normal tissue. In this respect, EGFR/HER2 signaling has been shown to induce a PKCδ-dependent phosphorylation of ERRα, leading to its stabilization in various breast cancer cell lines [43–44]. Strikingly, our pair-wise comparison revealed an increased LSD1 mRNA expression in tumors as compared to normal tissues. We assume that the corresponding protein follows this increase, in agreement with published data reporting LSD1 protein overexpression in breast cancers [12–14]. We propose that LSD1, through its stabilizing activity, is an essential factor in enhancing ERRα protein expression.

Our previous work has shown that, *in vitro*, ERRα induces LSD1 to demethylate the lysine 9 of histone 3 (H3K9), a phenomenon which results in transcriptional activation [34]. Consistently, a number of commonly up-regulated target genes has been identified, the promoter of which undergoes LSD1-ERRα-mediated H3K9 demethylation. Our bio-informatical analysis has shown that common LSD1-ERRα targets are considerably enriched in genes involved in the regulation of cell migration and invasion, which are hallmarks of aggressive cancers. Both aspects of the LSD1-ERRα relationships (activation of LSD1 by ERRα; stabilization of ERRα by LSD1) may thus contribute to increased regulation of downstream targets and thereby to cancer progression. Taken together, our results suggest that pharmacological targeting of LSD1 in aggressive cancers may not only inhibit the intrinsic activity of the demethylase but also decrease the level of ERRα protein. These effects could both contribute to the reduction of cancer aggressiveness.

## Supporting information

S1 Fig ERRα protein is stabilized by LSD1 in a proteasome dependent manner. **a.** Analysis of ERRα and LSD1 protein levels in HeLa cells at the indicated time after siLSD1 transfection. **b.** Analysis of ERRα and LSD1 protein levels in HeLa cells after the indicated siRNA transfection and treatment with MG132 or vehicle. **c.** Expression of the indicated genes analyzed by RT-qPCR in HeLa or MDA-MB231 cells after the indicated siRNA treatment, relative to control conditions. Values are presented as mean +/- sem of three independent experiments performed in triplicate. Significance was analyzed using Student t-test and is shown relative to control conditions. ***: *p*<0.005, ns: non significant. **d.** Same as Fig 2C, showing ubiquitylation of ERRα after treatment with an independent siRNA targeting LSD1. **e.** Co-immunoprecipitation of endogenous proteins with anti-ERRα antibody (or rabbit IgG as control) from HeLa cells treated with tranylcypromine (TCP) or vehicle (-) in the presence of MG132.
(TIFF)

S2 Fig Specificity of ERRα protection by LSD1. **a.** Detection of the indicated proteins in MDA-MB231 cells after treatment with siRNAs. Quantifications of AR and ERRα levels (relative to actin) are displayed. **b.** Co-immunoprecipitation of endogenous proteins with anti-AR or anti-ERRα antibody (or rabbit IgG as control) from MDA-MB231 cells. Note that the 100kDa AR isoform was detected and interacted with LSD1 in these cells. **c.** Detection of LSD1 in HeLa cells after treatment with siERRα. **d.** HeLa cells were transfected with the indicated flagged-ERRα derivatives (scheme, not to scale, displayed above; DBD: DNA-binding domain, LBD: ligand-binding domain) and treated with pargyline or vehicle.
(TIFF)
