# Supplementary figures and images for: ERRα protein is stabilized by LSD1 in a demethylation-independent manner
Source: PLoS One. 2017 Nov 30;12(11):e0188871. doi: 10.1371/journal.pone.0188871 (PMC5708767; doi:10.1371/journal.pone.0188871)

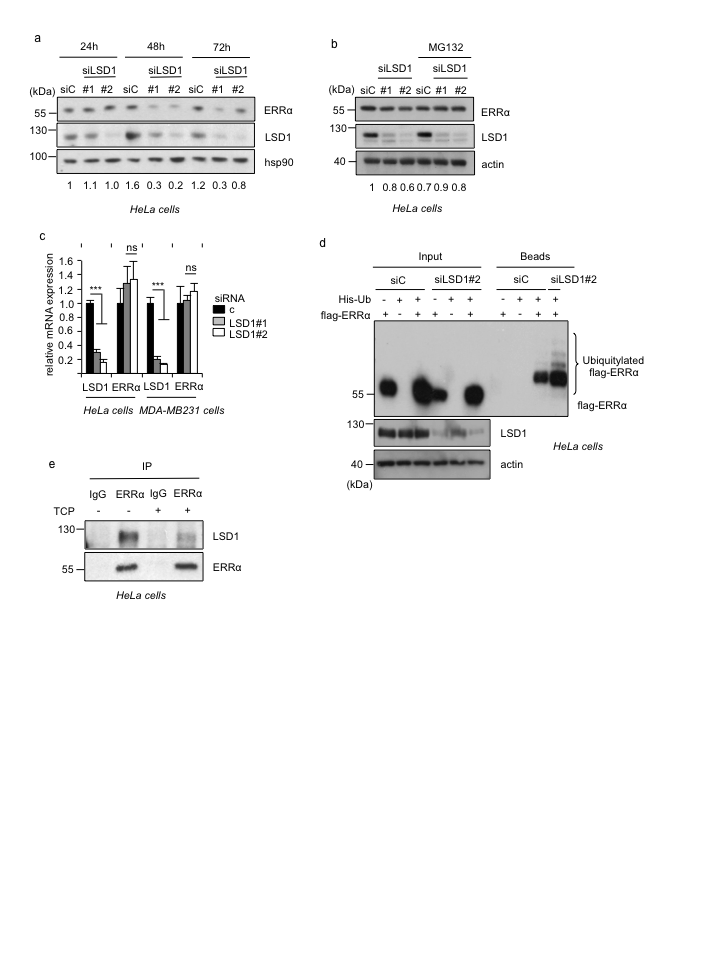

Supplement: S1 Fig — a. Analysis of ERRα and LSD1 protein levels in HeLa cells at the indicated time after siLSD1 transfection. b. Analysis of ERRα and LSD1 protein levels in HeLa cells after the indicated siRNA transfection and treatment with MG132 or vehicle. c. Expression of the indicated genes analyzed by RT-qPCR in HeLa or MDA-MB231 cells after the indicated siRNA treatment, relative to control conditions. Values are presented as mean +/- sem of three independent experiments performed in triplicate. Significance was analyzed using Student t-test and is shown relative to control conditions. ***: p<0.005, ns: non significant. d. Same as Fig 2C, showing ubiquitylation of ERRα after treatment with an independent siRNA targeting LSD1. e. Co-immunoprecipitation of endogenous proteins with anti-ERRα antibody (or rabbit IgG as control) from HeLa cells treated with tranylcypromine (TCP) or vehicle (-) in the presence of MG132. (TIFF) [file pone.0188871.s001.tiff]

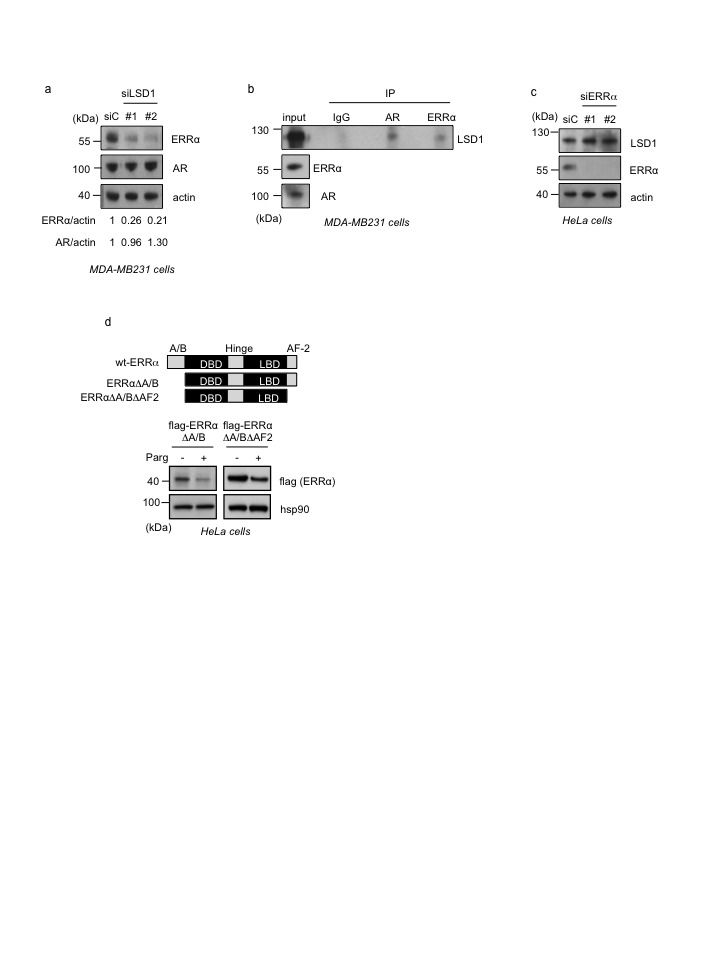

Supplement: S2 Fig — a. Detection of the indicated proteins in MDA-MB231 cells after treatment with siRNAs. Quantifications of AR and ERRα levels (relative to actin) are displayed. b. Co-immunoprecipitation of endogenous proteins with anti-AR or anti-ERRα antibody (or rabbit IgG as control) from MDA-MB231 cells. Note that the 100kDa AR isoform was detected and interacted with LSD1 in these cells. c. Detection of LSD1 in HeLa cells after treatment with siERRα. d. HeLa cells were transfected with the indicated flagged-ERRα derivatives (scheme, not to scale, displayed above; DBD: DNA-binding domain, LBD: ligand-binding domain) and treated with pargyline or vehicle. (TIFF) [file pone.0188871.s002.tiff]
